# Clinical outcome of intrauterine infusion of platelet‐rich plasma in patients with recurrent implantation failure

**DOI:** 10.1002/rmb2.12417

**Published:** 2021-09-30

**Authors:** Yihsien Enatsu, Noritoshi Enatsu, Kanako Kishi, Junko Otsuki, Toshiroh Iwasaki, Eri Okamoto, Shoji Kokeguchi, Masahide Shiotani

**Affiliations:** ^1^ Hanabusa Women’s Clinic Kobe city Japan; ^2^ Okayama University Assisted Reproduction Technology Center Okayama city Japan

**Keywords:** assisted reproductive technology, embryo implantation, endometrial thickness, platelet‐rich plasma, recurrent implantation failure

## Abstract

**Purpose:**

This study aimed to evaluate the effectiveness of intrauterine infusion of platelet‐rich plasma (PRP) before embryo transfer (ET) in recurrent implantation failure (RIF) cases.

**Methods:**

The authors retrospectively analyzed 54 ET cycles involving frozen and thawed high‐quality blastocysts after intrauterine PRP infusion between September 2019 and November 2020. All patients had a history of at least two times of implantation failure on ET. A total of 54 patients were categorized into two groups: thin endometrium (39 patients) and unexplained implantation failure (15 patients). In the thin‐endometrium group, the endometrial thickness (EMT) was <8.0 mm at cycle days 12–14 in the prior ET cycle.

**Results:**

Among the 54 ET cycles after PRP infusion, 31 (57.4%) were positive for human chorionic gonadotropin (hCG) and 27 (50%) achieved clinical pregnancy, which was significantly better than that in prior ET cycles without PRP infusion (27.2% and 9.6%, respectively). The EMT was not increased at ET date on the PRP cycle compared with that in the prior ET cycle in both patient groups. Moreover, EMT was not different between the hCG‐positive and hCG‐negative groups.

**Conclusion:**

Although intrauterine PRP infusion had no superior effect on increasing the EMT than conventional therapeutic agents, it resulted in high pregnancy rates in patients experiencing RIF with or without thin endometrium.

## 1 INTRODUCTION

Recurrent implantation failure (RIF) is an uncommon, vaguely defined clinical disorder characterized by unsuccessful pregnancies after repeated good‐quality embryo transfers (ETs). The etiologies of RIF are complex, attributing to multiple factors, such as thin endometrium, chronic endometritis, stress, immunological factors, incompatible implantation window, and specific autoantibodies.^1^ Among these factors, thin endometrium is an important factor in implantation failure, and it correlates with increased miscarriage risk.^2^, ^3^ The reason is pathophysiologically explained by poor epithelial growth, reduced expression of vascular endothelial growth factor (VEGF), poor vascular development, and the high impedance of blood flow in the radial arteries of the uterine vasculature.^4^ Although the endometrial thickness (EMT) reportedly improves through prolonged estrogen administration, aspirin, Vitamin E, and pentoxifylline, many women with thin endometrium remain nonresponsive with these treatments.^5^, ^6^ Platelet‐rich plasma (PRP) is a new modality that has recently been suggested for thin‐endometrium treatment.^7^, ^8^, ^9^ PRP is prepared from fresh blood that is collected from a peripheral vein and processed to increase the concentration of platelets by separating various blood components.^10^ Platelets contain a significant amount of growth factors, such as VEGF, epidermal growth factor, platelet‐derived growth factor (PDGF), and transforming growth factor, which all stimulate proliferation and growth.^11^ In the endometrium, angiogenesis is critical for endometrial growth after menstruation, and a vascularized receptive endometrium is essential for implantation. Therefore, growth factors and other cytokines found in PRP may promote endometrial thickening in patients with thin endometrium.^7^, ^12^

Until recently, intrauterine PRP infusion has been extensively reported to be effective in treating patients with a thin endometrium. According to Kusumi et al.,^8^ EMT was increased by 1.27 mm after PRP administration compared with the previous ET cycle. Similarly, several researchers inferred that PRP infusion may effectively improve the endometrial growth and possibly, pregnancy outcomes in women with thin endometrium.^7^, ^9^, ^13^ Nasari et al.^14^ also reported that PRP infusion improved the implantation rate in patients with RIF. However, reports on the effect of PRP infusion on women without thin endometrium are currently limited. Thus, we aimed to retrospectively investigate the effect of PRP infusion on patients with RIF and compare the outcomes between patients with RIF with thin endometrium and those without thin endometrium.

## 2 MATERIALS AND METHODS

This retrospective study included 54 cycles of frozen and thawed ET cycles performed after intrauterine PRP infusion at Hanabusa Women's Clinic between September 2019 and November 2020. The inclusion criteria of this study were patients with RIF, defined as having a history of at least two consecutive cycles of implantation failure and 25–45 years of age during ET. To exclude the influence of embryo quality, we only analyzed ETs with high‐quality blastocysts. Blastocysts were graded according to Gardner's classification,^15^ and a blastocyst grade of 3BB or better was considered as a high quality. The exclusion criteria were hepatic disorder, hemoglobin level <11 g/dl, platelet count <150 million/mm^3^, anticoagulant administration, and congenital uterine anomalies. This study was approved by the Certified Committee for Regenerative Medicine (accreditation No. NA8160002) and submitted to the Ministry of Health, Labour and Welfare in Japan (clinical study No. PB5190013). This study was also approved by our institutional ethics committee and was performed according to the ethical principles of the Declaration of Helsinki. All patients were well informed and provided written informed consent before the PRP infusion cycles.

**FIGURE 1:**
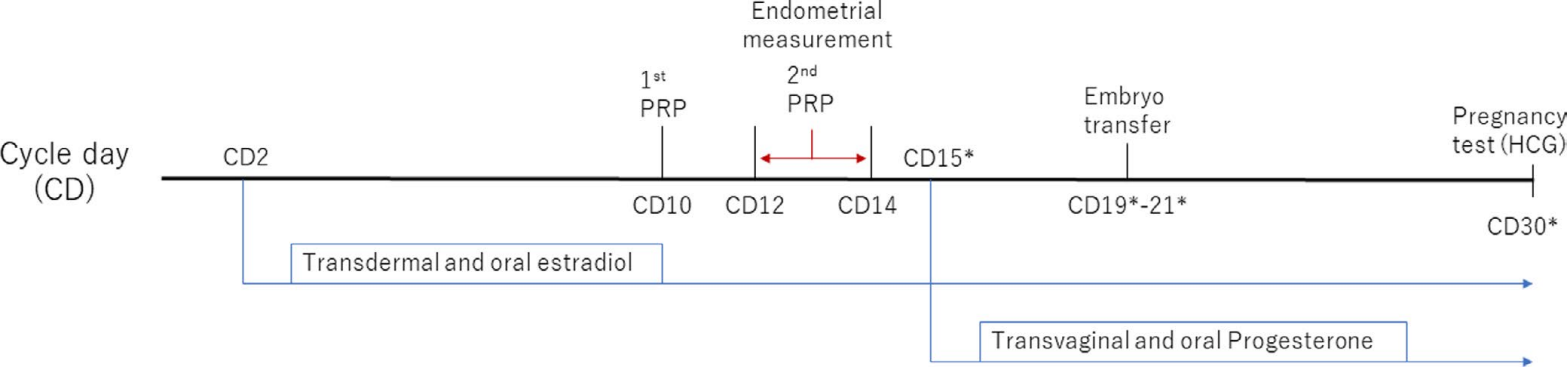
Timeline of intrauterine platelet‐rich plasma (PRP) infusion and embryo transfer in a hormone replacement cycle. *Cycle day (CD) 15 was adjusted as the date of the start of progesterone administration

Frozen and thawed ET was performed with a hormone replacement cycle, which was provided after ovarian suppression using a similar method reported previously.^16^ Figure 1 represents the timeline of the ET cycle protocol, including intrauterine PRP infusion. Administration of transdermal and oral estradiol was started on cycle day (CD) 2. The EMT was measured by transvaginal ultrasonography and intrauterine PRP infusion was performed on CD 10 (first time) and CDs 12–14 (second time). Patients with an EMT <8 mm on CDs 12–14 before the ET cycle were categorized as the thin‐endometrium group. The other patients were categorized as unexplained implantation failure. Some patients with thin endometrium who underwent prior ET cycles had received other conventional treatments such as prolonged estrogen, vitamin E, and pentoxifylline. These agents were not adapted in the PRP infusion cycle. We then compared the outcomes of prior ET cycles with those of the PRP cycles in the same patients. For this comparison, we only used the consecutive prior frozen and thawed ET cycles performed under the hormone replacement cycle with high‐quality blastocysts at the same CD. Cycles in which an additional therapy such as chronic endometritis therapy, endometrial polyp resection, vitamin D supplementation, or aspirin administration was given as well as cycles in which the endometrial receptivity analysis (ERA) test was performed until the PRP infusion cycles were excluded.

Platelet‐rich plasma was prepared from autologous peripheral blood using a similar method reported previously.^8^ Using two vacuum blood collection tubes (Acti‐PRP tube, Aeon International Inc.), we collected 20 ml of peripheral blood from the forearm. Then, the blood was centrifuged at 2000 *g* for 6 min and divided into three layers: bottom layer (containing red and white blood cells), supernatant layer (containing cellular plasma), and a buffy coat layer (located between the two layers). We obtained 1 ml (0.5 ml per tube) of PRP from the bottom of the supernatant layer. Immediately after collecting the PRP, we infused the entire PRP volume (1 ml) into the uterine cavity using a soft catheter (Kitazato Medical Co., Ltd.). Correct fluid infusion was confirmed by transvaginal ultrasonography. Transvaginal and oral progesterone was administered on CD 15; the date was adjusted as CD 15 even when the progesterone start date was changed. ET was performed basically on CD 20 (5 days after progesterone administration), but the date was changed in cases which the previous ERA test recommended another preferable date.

In this study, the primary outcome was clinical pregnancy. At CD 30 (10 days after ET), serum human chorionic gonadotropin (hCG) was evaluated; positive hCG was considered when the hCG level was >5 mIU/ml. Further, we defined clinical pregnancy as the presence of a gestational sac with heart beats in the uterus via transvaginal ultrasonography 3 weeks after the ET. The secondary outcome was the increase in EMT; the increments in EMT from CD 10 (first PRP infusion) to CD 14 (second PRP infusion) and CD 20 (ET date) were compared with those of the prior ET cycles without PRP infusion.

All statistical data were calculated using Student's *t*‐test or chi‐square test and analyzed using Excel (Office 365, Microsoft USA) and EZR (Saitama Medical Center), which is a graphical user interface for R (The R Foundation for Statistical Computing). Moreover, *p*‐values <0.01 were considered significant.

## 3 RESULTS

Table 1 summarizes the characteristics of 54 included patients, of which 15 constituted the unexplained implantation failure group and 39 belonged to the thin‐endometrium group. Age, number of prior ART cycles, number of prior clinical pregnancy, and number of spontaneous abortion were not significantly different between two groups. These factors were also not different between the hCG‐positive group and hCG‐negative group.

**TABLE 1:** Baseline characteristics of patients

	HCG positive		HCG negative		*p*‐value
	Mean	SD	Mean	SD	
Unexplained implantation failure (*n* = 15)					
Age	37	5.2	40	3.4	0.07
No. of prior ART cycles	3	4.3	4	1.8	0.37
No. of prior ET	4	1.9	4	2.8	0.19
No. of prior clinical pregnancy	0	1.6	0	0.5	0.16
No. of prior spontaneous abortion	0	1.6	0	0.5	0.16
Implantation failure due to thin endometrium (*n* = 39)					
Age	38	5.6	40	2.4	0.45
No. of prior ART cycles	3	6.2	3	6.5	0.34
No. of prior ET	4	2.4	2	2.2	0.06
No. of prior clinical pregnancy	2	1.2	1	1.1	0.2
No. of prior spontaneous abortion	1	1	1	1	0.4

Abbreviations: ART, assisted reproductive technology; ET, embryo transfer; HCG, human chorionic gonadotropin; SD, standard deviation.

Table 2 represents the changes in EMT between the PRP cycles and the prior ET cycles. Naturally, the EMT at CDs 12–14 was thinner in the thin‐endometrium group than in the unexplained implantation failure group; the EMT was almost the same in PRP infusion cycles and prior ET cycles in both groups. Regardless of the EMT at CDs 12–14, the endometrium did not thicken at the ET date on the PRP infusion cycles. When comparing the EMT between the hCG‐positive and hCG‐negative groups, no difference was found in both the unexplained implantation failure group and the thin‐endometrium group.

**TABLE 2:** Changes in endometrial thickness (mm) at PRP infusion cycles in comparison with prior ET cycle

	Overall		*p*‐value^a^	HCG positive		HCG negative		*p*‐value^b^	*p*‐value^a^
	mean	SD		mean	SD	mean	SD		
Unexplained implantation failure (*n* = 15)									
At CD 12–14 on prior ET cycle	8.6	1.5		8.7	1.2	8.3	1.6	0.38	
At ET date on prior ET cycle	9.1	1.7		9.1	1.4	9.3	1.7	0.27	
At first PRP infusion	7.5	1.1		7.6	1.3	7.2	0.8	0.31	
At second PRP infusion	8	1.4	0.37	8.1	1.8	8	1	0.27	0.30/0.31
At ET date on PRP cycle	9.1	0.9	0.43	9.3	0.9	9.1	0.9	0.45	0.41/0.22
Implantation failure due to thin endometrium (*n* = 39)									
At CD 12–14 on prior ET cycle	6.5	1		6.7	1	6.3	1	0.16	
At ET date on prior ET cycle	7.7	1		7.5	1.4	7.9	1.3	0.38	
At first PRP infusion	6.2	0.8		6.2	1.2	6.1	1.2	0.43	
At second PRP infusion	6.4	1.2	0.25	6.5	1.3	6.3	1	0.39	0.11/0.07
At ET date on PRP cycle	7.2	1.2	0.21	7.2	1	7.6	1.3	0.17	0.19/0.47

Abbreviations: CD, cycle day; ET, embryo transfer; HCG, human chorionic gonadotropin; PRP, platelet‐rich plasma; SD, standard deviation.

^a^ Comparison between prior ET cycle and PRP infusion cycle.

^b^ Comparison between the HCG‐positive and HCG‐negative groups.

Table 3 reveals the pregnancy outcomes of ET between the PRP cycles and the prior ET cycles. Overall, the hCG positivity rate and clinical pregnancy rate were significantly higher (57.4% and 50.0%) in the PRP cycles than in the prior ET cycles (27.2% and 9.6%). In addition, spontaneous abortion rate was less in PRP cycles (11.1%) than in prior ET cycles (55.5%), though the difference was not statistically significant because of the small sample size. Consequently, ongoing pregnancy (>15 gestational weeks) or live birth rate was significantly higher in the PRP cycles (44.4%) than in the prior ET cycles (4.3%). This tendency was similar between the unexplained implantation failure group and thin‐endometrium group.

**TABLE 3:** Pregnancy outcomes in patients who underwent frozen and thawed embryo transfer after PRP instillation in comparison with previous cycles. The analyses contain only high‐quality blastocyst transfer in hormone replacement cycles both in PRP cycles and prior ET cycles

Outcome	PRP cycles	Previous cycles	Odds ratio	*p*‐value
Overall	*n* = 54	*n* = 187		
HCG positive	31 (57.4%)	51 (27.2%)	3.6	<0.01
Clinical pregnancy	27 (50.0%)	18 (9.6%)	9.4	<0.01
Spontaneous abortion	3 (11.1%)	10 (55.5%)	0.2	0.07
Ongoing pregnancy or live birth	24 (44.4%)	8 (4.3%)	14	<0.01
Unexplained implantation failure	*n* = 15	*n* = 68		
HCG positive	8 (53.3%)	12 (17.6%)	5.3	<.01
Clinical pregnancy	6 (40.0%)	4 (5.9%)	10	<0.01
Spontaneous abortion	0 (0%)	3 (75.0%)	0	0.19
Ongoing pregnancy, live birth	6 (40.0%)	1 (1.5%)	27	<0.01
Implantation failure due to thin endometrium	*n* = 39	*n* = 119		
HCG positive	23 (59.0%)	39 (32.8%)	2.9	<0.01
Clinical pregnancy	21 (53.8%)	14 (11.8%)	8.8	<0.01
Spontaneous abortion	3 (14.3%)	7 (50.0%)	0.2	0.06
Ongoing pregnancy, live birth	18 (46.2%)	7 (5.9%)	13	<0.01

Abbreviations: ET, embryo transfer; HCG, human chorionic gonadotropin; PRP, platelet‐rich plasma.

## 4 DISCUSSION

Embryo implantation is a delicately coordinated event with multiple underlying factors. Although embryonic factor is the most important for pregnancy, many other factors were related to the in vitro fertilization outcomes. In fact, recent studies reported that <60% of euploid embryos resulted in ongoing pregnancy.^17^ Among many variables, optimal EMT is one of the critical factors related to a successful embryo implantation.^2^, ^3^ Until recently, intrauterine PRP infusion has been extensively reported to have a positive impact on EMT and pregnancy rate.^7^, ^8^, ^9^ Autologous PRP is developed from autologous blood and is, therefore, inherently safe and free of transmissible diseases, such as HIV and hepatitis. Based on the pioneering and long‐term clinical experience on the application of PRP in the oral‐maxillary field and thousands of patients having received this therapy so far, the use of PRP is considered safe.^18^ In this study, no adverse events, such as injury and infection, were observed. Nazari et al.^14^ conducted the largest randomized control trial, which included 138 patients with RIF, and revealed a significantly better clinical pregnancy rate in the PRP group than in the control group (44.89% vs. 16.66%). In this study, we investigated the effectiveness of the intrauterine PRP infusion at the ET cycle. Unexpectedly, we found that the EMT was not increased at the ET date by intrauterine PRP infusion compared with that in the prior cycles. The reason could be that we added other conventional therapeutic agents, such as estrogen, Vitamin E, and pentoxifylline in most cases with thin endometrium at prior ET cycles and did not add these agents in the PRP cycles in order to prevent unknown interactions between PRP and these agents. Moreover, we considered that prolonged estrogen administration might prolong the interval between PRP administration and ET, which may weaken the effects of PRP. Therefore, we first prioritized to follow the PRP schedule rather than waiting for the endometrium to become thick. In fact, the EMT at the prior ET cycle was higher at ET date than that at CDs 12–14, indicating that the other remedies, as well as PRP, had a positive impact on EMT until the ET date. Ledee et al.^19^ reported that combined treatment by pentoxifylline and tocopherol increased the EMT with a mean of 1.3 mm in the ET cycle, similar to the EMT increase in the present study (1.2 mm in the thin‐endometrium group). Interestingly, our study revealed that the EMT on ET date was not different between the hCG‐positive and hCG‐negative cycles; hence, factors other than EMT may play more important roles in embryo implantation. In addition, the PRP infusion cycles resulted in a higher pregnancy rate both in the thin endometrium and unexplained implantation failure groups. Gingold et al.^20^ reported that EMT was not significantly associated with the clinical outcomes of euploid ETs, but the endometrial pattern was associated with such outcomes. In similar studies, a triple‐line endometrial pattern before or after the ovarian stimulation day was associated with a better pregnancy rate than a homogenous, hyperechoic, or intermediate endometrial pattern.^21^, ^22^ Thus, endometrial receptivity should be evaluated using an endometrial pattern and not EMT. In contrast, a previous study reported that the endometrial pattern was not associated with the pregnancy rate in ET.^23^ These conflicting reports indicate that the endometrial capacity for implantation is difficult to be clearly assessed by ultrasonography, suggesting that invisible interaction such as molecular mechanism is more important.

Although the molecular mechanisms of PRP therapy in the endometrium are still poorly understood, numerous growth factors are associated with endometrial growth. Platelets contain many growth factors, including PDGF, transforming growth factor beta, VEGF, insulin‐like growth factor, and keratinocyte growth factor, as well as many cytokines, including interleukin (IL)‐4, IL‐8, IL‐13, IL‐17, tumor necrosis factor (TNF)‐alpha, and interferon‐alpha.^24^ With these growth factors and cytokines, PRP accelerates angiogenesis in wound and soft tissue, leading to rapid tissue regeneration.^25^ Thus, PRP has been widely used for regenerative medicine, such as tissue regeneration. Considering these characteristics, PRP with a high concentration of growth factors and cytokines can stimulate the mitogenesis and proliferation of endometrial cells and endometrial stem cells. It can also activate the endocrine‐paracrine pathways for improving the endometrial response to promote embryo implantation and pregnancy.

Whether the PRP infusion decreases the risk of early miscarriage is still unknown. In this study, we reported that intrauterine PRP infusion decreases the spontaneous abortion rate. As patients with thin endometria have been reported to have a significantly increased risk for early pregnancy loss,^26^ an insufficiently prepared endometrium supposed to be not capable to maintain pregnancy. Further analysis is needed to understand the mechanism by which PRP decreases the risk of spontaneous abortion. However, it could be said that the PRP infusion helped with endometria to obtain sufficient function to maintain pregnancy.

The main limitation of this study is its retrospective study design with a relatively small sample size. In addition, patient's recruitment depended on physician's and patient's preference; therefore, selection bias may occur. Moreover, the underlying mechanism and biological pathways are still unclear, especially for patients with unexplained implantation failure.

## CONFLICT OF INTEREST

Yihsien Enatsu, Noritoshi Enatsu, Junko Otsuki, Toshiro Iwasaki, Eri Okamoto, Shoji Kokeguchi, Masahide Shiotani declare that they have no conflict of interest.

## HUMAN RIGHTS STATEMENT AND INFORMED CONSENT

All patients were well informed and written informed consent was obtained prior to the treatment period.

## ANIMAL RIGHTS

This article does not contain any studies with animal subjects performed by the any of the authors.

## THE STATEMENT OF APPROVAL FROM INSTITUTIONAL REVIEW BOARD

All procedures in this study were in accordance with the ethical standards of the Ethical Committee in accordance with the ethical principles that have their origin in the Declaration of Helsinki 1964 and its later amendments. This study was approved by Ethical Committee of Hanabusa Women's Clinic consists of members chosen by our institute and third‐party medical institute (approval number; 2021‐05).
